# Recognition of Natural Products as Potential Inhibitors of COVID-19 Main Protease (Mpro): In-Silico Evidences

**DOI:** 10.1007/s13659-020-00253-1

**Published:** 2020-06-17

**Authors:** Rohan R. Narkhede, Ashwini V. Pise, Rameshwar S. Cheke, Sachin D. Shinde

**Affiliations:** 1 Department of Medicinal Chemistry, National Institute of Pharmaceutical Education and Research (NIPER), Raebareli, Lucknow, 226002 India; 2 Department of Pharmaceutical Chemistry, Dr. Rajendra Gode College of Pharmacy, Makapur, Maharashtra 443101 India; 3 Department of Pharmacology, Shri. R.D. Bhakta College of Pharmacy, Jalna, Maharashtra 431203 India

**Keywords:** nCoV-2019, COVID-19 main protease, Herbal remedies, Docking study, Druggability

## Abstract

**Abstract:**

SARS-CoV-2 (2019-nCoV) emerged in 2019 and proliferated rapidly across the globe. Scientists are attempting to investigate antivirals specific to COVID-19 treatment. The 2019-nCoV and SARS-CoV utilize the same receptor of the host which is COVID-19 of the main protease (Mpro).COVID-19 caused by SARS-CoV-2 is burdensome to overcome by presently acquired antiviral candidates. So the objective and purpose of this work was to investigate the plants with reported potential antiviral activity. With the aid of in silico techniques such as molecular docking and druggability studies, we have proposed several natural active compounds including glycyrrhizin, bicylogermecrene, tryptanthrine, β-sitosterol, indirubin, indican, indigo, hesperetin, crysophanic acid, rhein, berberine and β-caryophyllene which can be encountered as potential herbal candidate exhibiting anti-viral activity against SARS-CoV-2. Promising docking outcomes have been executed which evidenced the worthy of these selected herbal remedies for future drug development to combat coronavirus disease.

**Graphic Abstract:**

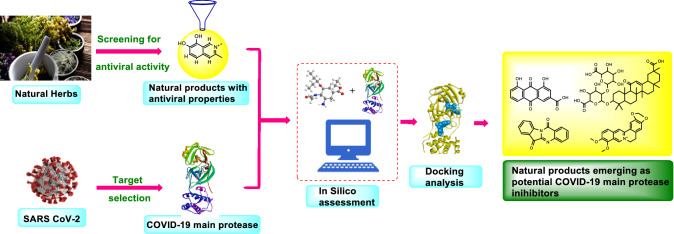

## Introduction

COVID-19 became a global risk to the healthcare system of almost every nation around the world. In the back of December 2019, a novel coronavirus strain was identified which was initially named as 2019 novel coronavirus (2019-nCoV) and it was evolved during an outbreak in Wuhan, Hubei province of China [1]. In China, a sudden outbreak was announced by The Emergency Committee of the World Health Organization (WHO) on 30th January, 2020 which was then regarded as Public Health Emergencies of International Concern [2]. Thereafter, the World Health Organization (WHO) has changed the name to coronavirus disease (COVID-19), on 11th February, 2020 [3]. According to the WHO situation report (107) around 35, 88, 773 cases of persons infected with coronaviruses (COVID-19) among which 2, 47, 503 death have been reported [4]. This virus is invading almost every nation of the world which puts a tremendous burden on the researchers and scientists worldwide to develop potential drug candidates to resist COVID-19.

There are no specialized treatments are available for the treatment of COVID-19 and several exploration relevant to the therapies of COVID-19 are becoming inadequate [2]. The available therapeutic options are limited to precautionary and provide support, employed for interception of additional consequences [2]. Few of the exploratory studies have encountered the combinations of existing drug candidates involving anti-HIV drugs such as lopinavir/ritonavir, remdesivir, arbidol, lamivudine tenofovir and disoproxil for therapeutic use against COVID-19 [5]. The recognition of protease as an attractive target to inhibit COVID-19 replication has emerged resulting in an investigation of drugs to target the viral protease [6–8].

Herbal plants provide a wide variety of integral and alternative medicine which may assist to solve the many puzzles behind many viral diseases [9]. Herbal remedies such as plant extract, plant-derived hybrid (phytoconstituents) herbal plant extract from specific parts of the plant (stem, roots, seed, barks, food and flower), nutraceuticals as well as nutritional supplements observe applications in treating disease vary from frequent to rare infectious and non-infectious ailments [10, 11]. A report of the World Health Organization (WHO), 80% of the human being in developing nations depends on traditional plants for health requirements [12, 13]. Many research has evidenced about plants derived products and their preparations promising tool against many viral infectious outbreaks [9]. Considering the low toxicity screening of herbal medicine [14] they can be employed extensively to target COVID-19 [15]. The main protease referred to as M^pro^ (3CL^pro^) recognized as a promising target among coronaviruses as it is primarily involved in the processing of viral polyproteins translated from viral RNA [6]. Since we have already investigated in-silico anti-COVID-19 activity of some potential drug candidates [16], in this mini-research we summarize the potential phytoconstituents that could target the main protease for treatment of nCoV-2019 by employing molecular docking tool. We proposed some natural products including glycyrrhizin, bicylogermecrene, tryptanthrine, β-sitosterol, indirubin, indican, indigo, hesperetin, crysophanic acid, rhein, berberine and β-caryophyllene as potential candidate for exerting the anti-viral activity against SARS-CoV-2 infection using molecular docking study.

Glycyrrhizin is abundantly found in dried roots of *Glycyrrhiza uralensis, Glycyrrhiza glabra*, and *Glycyrrhiza inflate* commonly known as licorice [17, 18]. Glycyrrhizin is known to exhibit potent antiviral activity [19, 20]. It is reported to elicit antiviral activity against Human Immunodeficiency Virus Type 1 (HIV-1) and Herpes Simplex Virus Type 1(HSV-1) [21], Hepatitis C virus [22], Varicella-Zoster virus [23] and SARS-Coronavirus [24]. Since licorice is an abundant and widely available medicinal plant since antiquity [25, 26], its potential antiviral activity may be attributed to COVID-19. Bicylogermecrene is a sesquiterpenoid [27] naturally occurring compound predominantly found in some species of eucalyptus [28]*, Lantana Camara*, and *Lantana* species and another plant including *Aloysia gratissima* belonging to family Verbenaceae [29]. Bicyclogermacrene has been delineated to exhibit various activities including antimicrobial activity antibacterial, antifungal, antiviral activity [29, 30]. Tryptanthrine and β-sitosterol and indirubin are the major chemical constituent present in *Strobilanthes cusia* leaf belonging to family Acanthaceae, predominantly found in India, Bangladesh, and Himalayan region [31, 32]. Tryptanthrine is reported to exhibit anti-Human coronavirus NL63 (HCoV-NL63) (IC_50_ 1.52 μM) which suggests the potent anti- HCoV-NL63 activity of it [32]. The other component which is indirubin is also reported to possess antiviral and immunomodulatory activity predominantly against influenza-A [33–35]. These natural products may act as a promising candidate to combat the COVID-19. Another potential natural product found in *S. cusia* leaf is β-sitosterol, a phytosterol which is recognized for its in *vitro* enzymatic inhibitory activity against SARS coronavirus 3C-like protease [36] and anti-HBV (hepatitis B virus) activity [37]. Besides this, it also exhibits antibacterial, anti-inflammatory and antitumor activities [38–40]. *Isatis indigotica* containing Indigo and indirubin is Chinese traditional medicine that is recognized as potential compounds for the treatment of hepatitis, and encephalitis SARS-coronavirus, influenza, foot-and-mouth disease, human immunodeficiency virus type 1 and rabies (HIV-1) [41–44]. Hesperetin is an effectual compound in Chenpi (*Citri Reticulatae Pericarpium)* belonging to family Rutaceae [45, 46] a product belonging to flavanone class of flavonoids is abundant in citrus fruits [47] have known to possess the anti-influenza viral activity and protective effect against fulminant hepatitis [47, 48]. Rhein and crysophanic acid are widely distributed in species of aloe vera (*Aloe barbadensis)* belonging to family Asphodelaceae [49] and Rhubarb (*Rheum palmatum)* belonging to family Polygonaceae [50] among which, crysophanic acid is reported to exhibit antiviral activity against poliovirus [51, 52] and rhein is known for its anti-influenza activity [51] and anti-Human Respiratory Syncytial Virus [50]. Berberine is an alkaloid of isoquinoline category which is abundant in *Berberis aristata* (Berberidaceae) and other species of berberis [52]. β-caryophyllene abundant in basil (*Ocimum* spp.), cinnamon (*Cinnamomum* spp.), black pepper (*Piper nigrum*), cannabis (*Cannabis sativa*), cloves (*Syzygium aromaticum*), oregano (*Origanum vulgare*), rosemary (*Rosmarinus officinalis*) and lavender (*Lavandula angustifolia*) which is already recognized to exhibit antiviral activity [53]. Berberine is widely accepted for its potential wide spectrum activity against a variety of viruses including Human Cytomegalovirus [54], influenza A/FM1/1/47 (H1N1) [55], enterovirus 71 (EV71) [52], Respiratory Syncytial Virus [56], Chikungunya virus (CHIKV) [57] and herpes simplex virus [58]. Thus, in this work, we have brought some phytoconstituents into play by demonstrating their in-silico anti-COVID-19 activity attributed by inhibiting the main protease by utilizing molecular docking tools.

## Result, Discussion and Conclusion

**Fig. 1:**
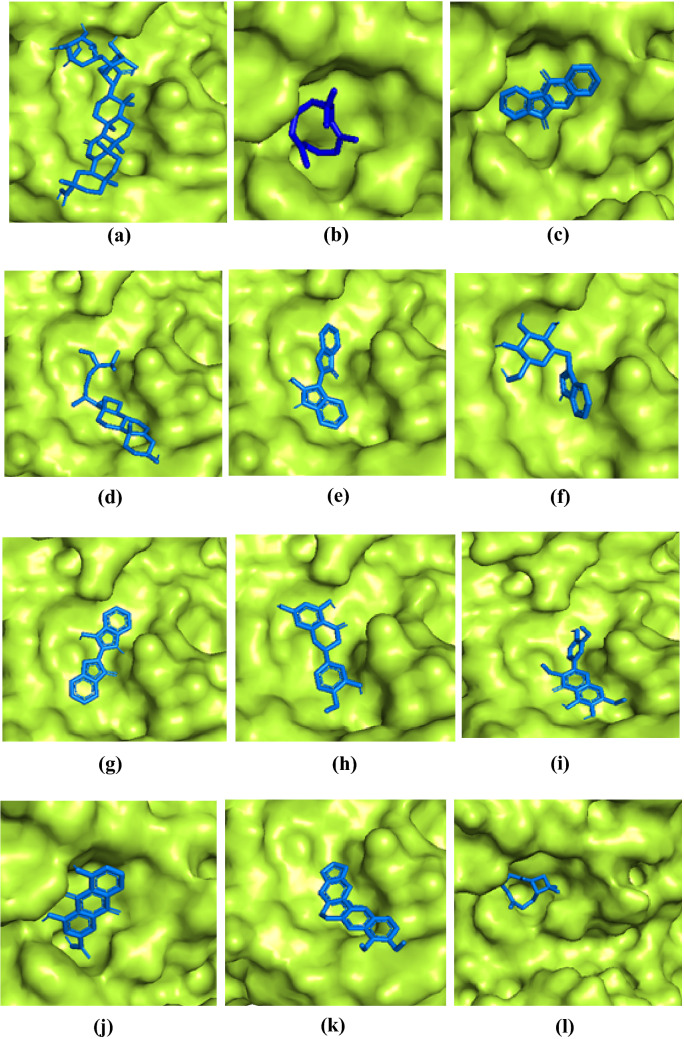
Visualization of docking results showing binding of **a** glycyrrhizin, **b** bicylogermecrene, **c** tryptanthrine, **d** β-sitosterol, **e** indirubin, **f** indican, **g** indigo, **h** hesperetin, **i** crysophanic acid, **j** rhein, **k** berberine and **l** β-caryophyllene inside the pocket of COVID-19 main protease with ligand as blue color sticks

**Fig. 2:**
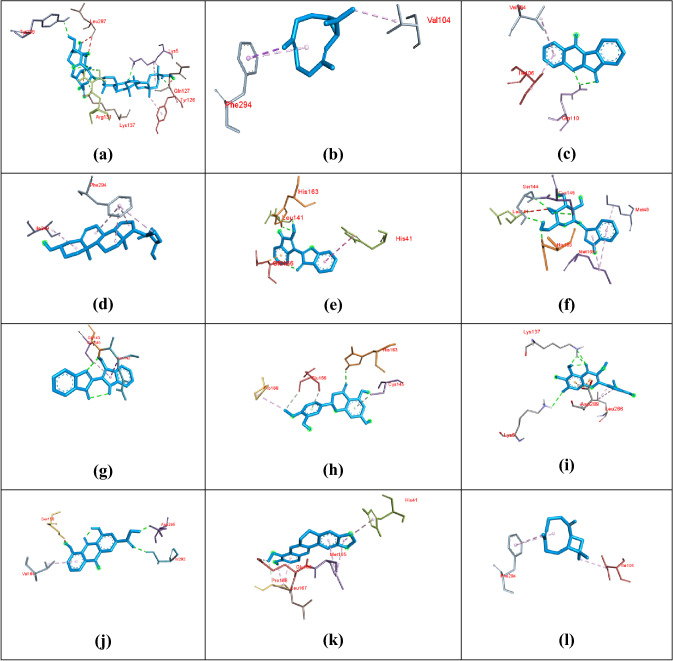
Visualization of docking results: amino acid residues involved in interaction of **a** glycyrrhizin, **b** bicylogermecrene, **c** tryptanthrine, **d** β-sitosterol, **e** indirubin, **f** indican, **g** indigo, **h** hesperetin, **i** crysophanic acid, **j** rhein, **k** berberine and **l** β-caryophyllene with COVID-19 main protease with ligand as blue color sticks and hydrogen bonding by green dash

After the docking experiment, a divergent poses of ligand were generated among which they pose with the best affinity (lowest in terms of kcal/mol) was considered as the best pose and further processed for visualization. The results acquired after docking analysis in terms of ligand binding affinity (kcal/mol), the interaction of natural products with the COVID-19 main protease, and the drug-like properties were shown in (Table 1). The drug-like properties were evaluated by Lipinski rule of five suggesting that most of the natural products were found to have no violations which can be attributed to their drug-likeness. The docking of the phytoconstituents in COVID-19 main protease was subsequently visualized for their binding in the pocket of the protein. The phytoconstituents displaying binding in the active site (pocket) of protein (represented as surface) is shown in (Fig. 1). The accommodation of phytoconstituents in the main protease revealed various amino acid residues engaged in interaction, are shown in (Fig. 2). These interactions were attributed to evidence of the in-silico ligand–protein interaction.

**Table 1:** Docking results of natural products in terms of binding affinity (kcal/mol), interaction of natural products with the COVID-19 main protease (PDB ID: 6LU7) and the drug like properties

Natural products	Affinity (kcal/mol)	Structure of compounds and its interaction with COVID-19 main protease	Drug like properties (Lipinski’s rule of five)
Glycyrrhizin	− 8.1		Molecular weight (< 500 Da): 822.93 Log P (< 5): 1.55 H-bond donor (5): 8 H-bond acceptor (< 10): 16 MlogP (< 4.15): 0.02 Violations: 3
Bicylogermecrene	− 6.5		Molecular weight (< 500 Da): 204.35 Log P (< 5): 4.15 H-bond donor (5): 0 H-bond acceptor (< 10): 0 MlogP (< 4.15): 4.63 Violations: 1
Tryptanthrine	− 8.2		Molecular weight (< 500 Da): 248.24 Log P (< 5): 2.16 H-bond donor (5): 0 H-bond acceptor (< 10): 3 MlogP (< 4.15): 2.22 Violations: 0
β-sitosterol	− 7.2		Molecular weight (< 500 Da): 414.71 Log P (< 5): 7.19 H-bond donor (5): 1 H-bond acceptor (< 10): 1 MlogP (< 4.15): 6.73 Violations: 1
Indirubin	− 7.6		Molecular weight (< 500 Da): 260.26 Log P (< 5): 2.69 H-bond donor (5): 2 H-bond acceptor (< 10): 3 MlogP (< 4.15): 1.70 Violations: 0
Indican	− 7.5		Molecular weight (< 500 Da): 295.29 Log P (< 5): − 0.16 H-bond donor (5): 5 H-bond acceptor (< 10): 6 MlogP (< 4.15): − 1.13 Violations: 0
Indigo	− 7.5		Molecular weight (< 500 Da): 262.26 Log P (< 5): 2.63 H-bond donor (5): 2 H-bond acceptor (< 10): 3 MlogP (< 4.15): 1.23 Violations: 0
Hesperetin	− 7.9		Molecular weight (< 500 Da): 302.28 Log P (< 5): 1.91 H-bond donor (5): 6 H-bond acceptor (< 10): 6 MlogP (< 4.15): 0.41 Violations: 0
Crysophanic acid	− 7.3		Molecular weight (< 500 Da): 254.24 Log P (< 5): 2.38 H-bond donor (5): 2 H-bond acceptor (< 10): 4 MlogP (< 4.15): 0.92 Violations: 0
Rhein	− 8.9		Molecular weight (< 500 Da): 284.22 Log P (< 5): 1.48 H-bond donor (5): 3 H-bond acceptor (< 10): 6 MlogP (< 4.15): 0.29 Violations: 0
Berberine	− 8.1		Molecular weight (< 500 Da): 336.36 Log P (< 5): 2.53 H-bond donor (5): 0 H-bond acceptor (< 10): 4 MlogP (< 4.15): 2.19 Violations: 0
β-caryophyllene	− 7.2		Molecular weight (< 500 Da): 204.35 Log P (< 5): 4.24 H-bond donor (5): 0 H-bond acceptor (< 10): 0 MlogP (< 4.15): 4.63 Violations: 1

The molecular docking revealed principle interactions that are transpiring between the natural products and the main protease of COVID-19. Since the native inhibitor (N2) accommodated in the crystal structure of COVID-19 main protease, it allowed the natural products to bind in the active sites of the protein. The active site analysis revealed the principle amino acid residues that are associated with ligand binding including PRO 168, ALA 191, THR 190, GLU 166, GLN 189, MET 49, ARG 188, HIS 41, ASP 187, HIS 164, CYS 145, GLY 143, THR 26, THR 24, THR 25, SER 144, MET 165, ASN 142, HIS 163, HIS 172, GLN 192, LEU 141 and PHE 140.

Glycyrrhizin showed a prominent interaction with the main protease accompanied by an affinity of − 8.9 kcal/mol and five hydrogen bonds specifically with GLN 127, LYS 5, LYS 137, ARG 131 and TYR 239. However, it is quite unfavorable to follow Lipinski's rule of five as it is characterized by three violations (Table 1). Bicylogermecrene binds with the main protease with an affinity of − 6.5 kcal/mole by establishing pi-sigma interactions with PHE 294. The docking of tryptanthrine with main protease is accompanied by an affinity of − 8.2 kcal/mol by forming two hydrogen bonds with GLN 110 along with pi-alkyl interactions with ILE 106, VAL 104 and several van der Waals interaction with ARG 105, GLN 107, THR 111, THR 292, PHE 294, ASP 153 and SER 158. It shows favorable drug-like properties by following Lipinski's rule of five without any violation. A significant binding of β-sitosterol with the target protein was observed with an affinity of − 7.2 kcal/mol designated by four pi-alkyl interaction with PHE 294. A desirable binding of indirubin and indican with the main protease was observed with an affinity of − 7.6 and − 7.5 respectively. These interactions are accompanied by three hydrogen bonds of indirubin specifically with HIS 163, LEU 141, GLU 166 and pi–pi interaction with HIS 41, and hydrogen bonds of indican with CYS 145, SER 144 and HIS 163. Both indirubin and indican exhibit drug-likeness as they were found to obey Lipinski's rule of five. Indigo was found to exhibit a hydrogen bond with GLY 143 and residue of the main protease with an affinity of − 7.5 kcal/mol. Indigo is also attributed to drug-like properties without any violation and possesses a strong potential to become a promising drug candidate. The interaction of hesperetin and crysophanic acid with the main protease was found to be prominent with an affinity of − 7.9 and − 7.3 kcal/mol and both natural products were found to be druggable. Hesperetin and crysophanic acid are accompanied by hydrogen bond with HIS 163 and LYS 137, LYS 5 respectively. A promising binding to the COVID-19 main protease was observed in the case of rhein and berberine where both natural products were found to exhibit an affinity of − 8.9 and − 8.1 kcal/mol respectively. Their binding is also accompanied by hydrogen bonds as rhein was manifested by two hydrogen bonds with ASO 295 and THR 292 along with van der Waals interaction with several amino acid residues including ASP 153, THR 111, ILE 106, GLN 110 and LYS 102. Similarly, berberine was also found to show carbon-hydrogen bonding with LEU 167, GLU 166, and pi-alkyl interaction with HIS 41 and MET 165. Both rhein and berberine was found to be druggable. Pie-alkyl interactions with PHE 294 were observed in the case of β-caryophyllene with an affinity of − 7.2. It was also found to exhibit drug-like properties with a violation of Lipinski's rule of five. The interactions with the COVID-19 main protease were highest in the case of glycyrrhizin and rhein indicating their potential to bind the protease which is evidenced by their efficient accommodation inside the pocket of the protein. This may provide a promising basis for their development as potential candidates to inhibit viral protease.

Since most of the drug candidates presently available for COVID-19 substantially act on viral main protease, by using molecular docking analysis, we have predicted the protease inhibitor activity of several natural products that can emerge as potential drug candidates inhibiting viral protease. A promising binding of natural products with the COVID-19 main protease was revealed by docking analysis. Among the several natural products screened by docking analysis, glycyrrhizin, tryptanthrine, rhein, and berberine were found to exhibit a higher degree of interaction with the viral protease accompanied by lowest binding energy with favorable drug-like properties. Thus these natural products may emerge as potential COVID-19 main protease inhibitor. However, additional exploration is inevitable for the investigation of the inherent use of the herbs containing these natural products and their *in-vivo* activity.

## Experimental Section

### Method

The molecular docking experiment of natural products was designed using COVID-19 main protease in complex with an inhibitor N3 [59] (PDB ID: 6LU7) as macromolecule.

### Retrieval of Target Protein

The protein structure COVID-19 main protease in complex with an inhibitor N3 [59] (PDB ID: 6LU7) was retrieved from the protein data bank (https://www.rcsb.org/pdb/home/home.do). The structure was downloaded in PDB format and the inhibitor N3 [59] from the protein structure was removed. The protein was prepared by removing the water molecules followed by adding Kollman charges and polar hydrogen’s and saved in PDBQT format for docking analysis.

### Retrieval of Ligand

The 3D structures of the natural products were obtained from PubChem Database. Respective CIDs of the compounds for Pubchem database are follows; glycyrrhizin (CID:128229, bicylogermecrene (CID:13894537), tryptanthrine (CID:73549), β-sitosterol (CID:222284), indirubin (CID:10177), indican (CID:441564), indigo (CID:10215), hesperetin (CID:72281), crysophanic acid (CID:10208), rhein (CID:10168), berberine (CID:2353) and β-caryophyllene (CID: 5281515). ChemDraw 12.0 was used to generate 2D structures of these ligands [60]. The ligands were energetically minimized by the use of the Vega ZZ program [61] along with the SP4 force field and conjugate gradient method.

### Docking Experiment

Auto Dock Vina was employed as a program for docking experiments using an exhaustiveness value of 80 using Auto dock Vina 1.0 [62]. The fixing of the grid box at the active site of protein was performed using Auto Dock Tools 1.5.6 [63]. The grid was set using x, y, z of 40, 40, 40, and x, y, z center as − 26.283, 12.599, 59.154 respectively. For docking, the final ligand and protein were prepared using Auto Dock tools 1.5.6. The remaining parameters of the program were kept as default considering movable ligand and rigid protein. The docking result was visualized using Pymol 1.8.6.0 and Discovery Studio Visualizer 4.0 [64]. The assessment of the natural products for drug-like properties was accomplished by Lipinski’s rule of five [65, 66]. The drug-like properties accommodated in Lipinski's rule of five were calculated by employing the Swiss ADME web tools [67].
